# Theoretical and empirical comparisons of expected and realized relationships for the X-chromosome

**DOI:** 10.1186/s12711-020-00570-6

**Published:** 2020-08-20

**Authors:** Tom Druet, Andres Legarra

**Affiliations:** 1 Unit of Animal Genomics, GIGA-R & Faculty of Veterinary Medicine, University of Liège, Liège, Belgium; 2 GenPhySE, INPT, INRAE, ENVT, 31326 Castanet Tolosan, France

## Abstract

**Background:**

X-chromosomal loci present different inheritance patterns compared to autosomal loci and must be modeled accordingly. Sexual chromosomes are not systematically considered in whole-genome relationship matrices although rules based on genealogical or marker information have been derived. Loci on the X-chromosome could have a significant contribution to the additive genetic variance, in particular for some traits such as those related to reproduction. Thus, accounting for the X-chromosome relationship matrix might be informative to better understand the architecture of complex traits (e.g., by estimating the variance associated to this chromosome) and to improve their genomic prediction. For such applications, previous studies have shown the benefits of combining information from genotyped and ungenotyped individuals.

**Results:**

In this paper, we start by presenting rules to compute a genomic relationship matrix (GRM) for the X-chromosome (**G**^X^) without making any assumption on dosage compensation, and based on coding of gene content with 0/1 for males and 0/1/2 for females. This coding adjusts naturally to previously derived pedigree-based relationships (**S**) for the X-chromosome. When needed, we propose to accommodate and estimate dosage compensation and genetic heterogeneity across sexes via multiple trait models. Using a Holstein dairy cattle dataset, including males and females, we then empirically illustrate that realized relationships (**G**^X^) matches expectations (**S**). However, **G**^X^ presents high deviations from **S**. **G**^X^ has also a lower dimensionality compared to the autosomal GRM. In particular, individuals are frequently identical along the entire chromosome. Finally, we confirm that the heritability of gene content for markers on the X-chromosome that are estimated by using **S** is 1, further demonstrating that **S** and **G**^X^ can be combined. For the pseudo-autosomal region, we demonstrate that the expected relationships vary according to position because of the sex-gradient. We end by presenting the rules to construct the '**H** matrix’ by combining both relationship matrices.

**Conclusions:**

This work shows theoretically and empirically that a pedigree-based relationship matrix built with rules specifically developed for the X-chromosome (**S**) matches the realized GRM for the X-chromosome. Therefore, applications that combine expected relationships and genotypes for markers on the X-chromosome should use **S** and **G**^**X**^.

## Background

Additive relationships and the associated matrices are important in essential applications such as estimation of the heritability of a complex trait, prediction of genomic values or inference of unknown relationships (e.g., in wild populations). The additive relationships can be estimated from pedigree data when the genealogy is available as in many livestock species. Alternatively, they can be inferred from genotypes at a set of markers. This requires the genotyping of the individuals but provides realized relationships contrary to pedigree-based estimators that are expected values. More accurate predictions are obtained when using genomic information. In addition, such genomic relationships are not affected by pedigree errors and can even be obtained without pedigrees. For these reasons, genomic relationships are superior to pedigree-based values, e.g. [1]. When only a subset of individuals is genotyped and the genealogy is available for other individuals, it might be advantageous to combine both relationship matrices [2]. This is, for instance, the core of single-step genomic best linear unbiased prediction (SSGBLUP) that results in higher prediction accuracies than GBLUP [2, 3]. The same strategy has also been applied in genome-wide association studies (GWAS), e.g. [4]. However, both approaches require that the genomic relationship is scaled appropriately.

The X-chromosome has often been ignored [5], and is still not systematically used (but see [6, 7]), in quantitative genetics applications (genetic or genomic predictions, GWAS) although it might have important contributions to the genetic variance since it is one of the longest chromosomes (in cattle) and this chromosome is gene rich [7–9]. The contribution of the X-chromosome to phenotypic variation might be important for fertility or reproduction traits, e.g. [10–12]. Examples include variants that affect litter size in sheep, e.g. [13], infertility in cattle [14], or bull fertility [11]. More generally, quantitative trait loci (QTL) on the X-chromosome have been reported in previous studies, e.g. [15], which indicates that this chromosome should be taken into account.

Fernando and Grossman [16] presented the rules to construct the X-chromosome relationship matrix $\mathbf{S}$ and its inverse from pedigree records, in a proper quantitative genetics framework. Contrary to popular belief, this matrix is quite different from its autosomal counterpart $\mathbf{A}$ (the “numerator relationship matrix”). Two important differences are that the diagonal values of $\mathbf{S}$ for males are always 0.5, and that there is no relationship between a male and its sire. For instance, the correlation of the off-diagonal elements of $\mathbf{A}$ and $\mathbf{S}$ across the last five generations (with complete pedigree tracing back to 40 generations) of line A in Fernandez et al. [17] is 0.79 across all females but only 0.36 across males (own calculation, results not shown). Still, in the pre-genomic era, relationships at the X-chromosome have been basically ignored.

In genomic analyses, the X-chromosome presents some specific challenges such as more complex inheritance patterns, lower quality of the genome assembly, lower genotype quality (lower call rate) and fewer markers on the arrays (see [5]). Rules to construct the genomic relationship matrix (GRM) have been proposed [7, 18] but they impose hypotheses such as the presence or absence of dosage compensation, yet dosage compensation varies across traits and tissues [8]. Although dosage compensation is not relevant for sex-specific traits such as milk or egg production, it should be estimated when the setting allows it (e.g., for phenotypes expressed in both sexes). In this paper, we illustrate that this can be achieved in a multiple trait setting. Then, we show that gene content for markers on the X-chromosome can be considered as a quantitative trait of heritability 1, naturally leading to applications that combine expected and realized relationships such as the single-step methods. We also provide rules to construct an $\mathbf{H}$ matrix for the X-chromosome combining genotyped and ungenotyped animals, either with metafounders or not.

Subsequently, we use real cattle data to verify whether the proposed pedigree-based and genomic relationships for the X-chromosome have similar expectations. In particular, we illustrate that genomic relationships are close to the expected values, and that the strong associated variation is due to the smaller size of the X-chromosome compared to all the autosomes considered together. To further illustrate the equivalence between the two relationship matrices, we estimate the heritability of the gene content [19] of markers located on the X-chromosome, and we show that it works as expected—heritability of gene content is equal to 1 when using pedigree relationships for markers on the X-chromosome, but is lower when using pedigree relationships for autosomal loci, because the latter does not describe correctly relationships for the X-chromosome.

Overall, this work shows theoretically and empirically that $\mathbf{S}$ matches the realized GRM on the X-chromosome. This allows extension of applications that combine expected relationships and genotypes to markers on the X-chromosome.

## Theory

Here, we briefly review the current theory for pedigree-based relationships [16] and marker-based relationships [7, 18] and suggest some extensions. We want to present a theory for the X-chromosome that, by construction, is compatible with existing pedigree-based methods. In general (unless explicitly mentioned), we will refer to the X-specific part. When we refer to the pseudo-autosomal region (PAR), we will use the abbreviation PAR. Our presentation will focus on mammals but concepts can be easily translated to birds reversing the sexes.

### Theory at a single-locus

Fernando and Grossman [16] derived rules to estimate a pedigree-based genetic relationship matrix ($\mathbf{S}$) for the X-chromosome. For a biallelic locus (e.g., *A*/*B*), males carry a single copy (coming from their dam) whereas females carry two copies (coming from their sire and dam). In their model, allelic effects were identical in males and females. This corresponds to defining the effects of loci in terms of expected differences across female descendants, but also to absence of dosage compensation (see definition below). Nevertheless, this relationship matrix can be rescaled to account for different levels of dosage compensation. As a result, it can be used for all traits and genetic architectures. Defining effects in female descendants is convenient as these represent an adequate reference population to define additive genotypic values and receive gametes from both their sire and dam. In addition, Fernando and Grossman [16] assumed no imprinting—this means that females receiving alleles *A* and *B* (from the sire and dam, respectively) will have the same genotypic value than females receiving alleles *B* and *A* (from the sire and dam, respectively). In this paper, we followed their hypotheses, including the absence of imprinting. By doing this, genomic and pedigree-based relationships will be compatible and coherent.

For a biallelic locus on the X-chromosome, numerical coding as gene content may proceed as {0,1} for males (say, for genotypes {*A*,*B*}) and {0,1,2} for females (say, for genotypes {*AA*,*AB*,*BB*}). This coding is consistent with the theory of Fernando and Grossman [16], and corresponds to the number of biological copies in males (that are hemizygotes). Note that equivalent GRM can be obtained using the {0,2} coding for males, proposed for instance by Su et al. [7], combined with appropriate scaling factors.

Imagine the generation n constituted by females (our reference population); sires from generation n-1 have genotypes either *A* or *B*. Because sires are haploids, their respective additive genotypic values (measured as expected progeny differences in females) are either $u=\left(1-p\right)\alpha$ or $u=-p\alpha$ where $p=freq(A)$, $q=freq(B)$ and $\alpha$ is the substitution effect of “*A*” in the female offspring. This is the reason we code gene content as {1,0} (for genotypes *A* and *B*, respectively) as proposed above. Accordingly, variance for gene content in males is $pq$ whereas variance for gene content in females is $2pq.$

For a given locus, gene content from all individuals can be summarized by a vector ${\mathbf{m}}^{\mathrm{X}}$ (hereafter, X refers to the X-chromosome) that contains the number of copies of the reference allele, {0,1} for males and {0,1,2} for females. The gene content at all loci can be encoded in a matrix ${\mathbf{M}}^{\mathrm{X}}$, with columns ${\mathbf{m}}_{j}^{\mathrm{X}}$ that contain the gene dosage at locus $j$, and elements ${M}_{i,j}^{\mathrm{X}}$ corresponding to the gene content of individual $i$ at that locus.

### Genomic relationships for the X-chromosome

From the above, and extending the reasoning to several marker effects in vector ${\varvec{\upalpha}}$, the additive genotypic value of individual $i$ in a female population would be ${u}_{i}={\mathbf{M}}_{\left[i,\right]}^{\mathrm{X}}{\varvec{\upalpha}}-E\left({\mathbf{M}}_{\left[i,\right]}^{\mathrm{X}}{\varvec{\upalpha}}\right)$ where ${\mathbf{M}}_{\left[i,\right]}^{\mathrm{X}}$ is the i-th row in matrix ${\mathbf{M}}^{\mathrm{X}}$. Then, we have:

$$\left(\begin{array}{c}{\mathbf{u}}_{males}\\ {\mathbf{u}}_{females}\end{array}\right)=\left(\begin{array}{c}{\mathbf{M}}_{males}^{\mathrm{X}}-1{\mathbf{p}}^{^{\prime}}\\ {\mathbf{M}}_{females}^{\mathrm{X}}-2{\mathbf{p}}^{^{\prime}}\end{array}\right){\varvec{\upalpha}}=\left(\begin{array}{c}{\mathbf{Z}}_{males}^{\mathrm{X}}\\ {\mathbf{Z}}_{females}^{\mathrm{X}}\end{array}\right){\varvec{\upalpha}}={\mathbf{Z}}^{\mathrm{X}}{\varvec{\upalpha}}$$

Note that the notion of dosage compensation does not intervene here because the male additive genotypic values are expressed on the trait in the female population. Using ${\sigma }_{u}^{2}=2\sum {p}_{i}{q}_{i}{\sigma }_{\alpha }^{2}$(in other words, we refer relationships to genetic variance in an ideal female population) we obtain:

$$Var\left( {\begin{array}{*{20}c} {{\mathbf{u}}_{males}} \\ {{\mathbf{u}}_{females}} \\ \end{array} } \right) = \frac{{\left( {\sigma _{u}^{2} } \right)}}{{\left( {2\sum {p_{i} q_{i} } } \right)}}\left( {\begin{array}{*{20}c} {{\mathbf{M}}_{males}^{X} - 1{\mathbf{p}^{\prime}}} \\ {{\mathbf{M}}_{females}^{X} - 2{\mathbf{p}^{\prime}}} \\ \end{array} } \right)\left( {\begin{array}{*{20}c} {{\mathbf{M}}_{males}^{X} - 1{\mathbf{p}^{\prime}}} \\ {{\mathbf{M}}_{females}^{X} - 2{\mathbf{p}^{\prime}}} \\ \end{array} } \right)^{\prime } = \frac{{\left( {{\mathbf{Z}}^{{\text{X}}} {\mathbf{Z}}^{{{\text{X}^{\prime}}}} } \right)}}{{\left( {2\sum {p_{i} q_{i} } } \right)}}\sigma _{u}^{2} = {\mathbf{G}}^{X} \sigma _{u}^{2} .$$

This is almost identical to the treatment of the chromosome X in Yang et al. [18] but we do not use standardized genotypes. This is also similar, but not identical, to VanRaden’s [20] ${\mathbf{G}}$ for autosomes. The differences between ${\mathbf{G}}$ and ${\mathbf{G}}^{{\text{X}}} { }$ are that, in the latter, males are coded as {0,1} and centered by $p$, not by $2p$, and the denominator refers to the genetic variance of a female population. The most important difference, that is not obvious in this matrix formulation, is that gene content for markers on the X-chromosome does not behave like gene content for markers on autosomes, even in females, because the paternal copy comes from the sire with no possibility of Mendelian sampling or recombination. This has implications that we will see later.

#### Some properties of the X-chromosome genomic relationship matrix

In a population with allele frequencies $p$, the average value of the diagonal elements of ${\mathbf{G}}^{{\text{X}}}$ is, as expected, 0.5 for males, but there are deviations from this value. By definition, there are no deviations from Hardy–Weinberg equilibrium or inbreeding in the male population, because there are no diploids—only haploids. Consider the diagonal elements $G_{i,i}^{{\text{X}}} = \frac{1}{W}\sum \left( {M_{i,j}^{X} - p_{j} } \right)^{2}$, $W = 2\sum p_{j} q_{j}$. In a population, for a given locus $j$, there is a proportion $p$ of males with genotypes $M_{i,j}^{X} = 1$ and a proportion 1*-*$p$ with genotypes $M_{i,j}^{X} = 0$. Weighting each square term by its probabilities, we obtain:

$$E\left( {G_{i,i}^{{\text{X}}} } \right) = \frac{1}{W}\sum \left[ {\left( {1 - p_{j} } \right)^{2} p_{j} + \left( {0 - p_{j} } \right)^{2} \left( {1 - p_{j} } \right)} \right]= \frac{1}{W}\sum \left[ {q_{j}^{2} p_{j} + p_{j}^{2} q_{j} } \right] = \frac{1}{W}\sum \left[ {\left( {q_{j} + p_{j} } \right)q_{j} p_{j} } \right] = \frac{1}{W}\sum p_{j} q_{j} = 0.5.$$

However, there are individual variations around this value, e.g. if some animals carry rare alleles. Using analogous arguments, it can be shown that the average value for females is 1, and that averages of ${\mathbf{G}}^{{\text{X}}}$ for a population of males, females, or both, are 0. Elements of ${\mathbf{G}}^{{\text{X}}}$ are comparable to pedigree relationships in ${\mathbf{S}}$ (Fernando and Grossman [16]) only if base-population allele frequencies are used. Otherwise, the matrix is biased (generally, relationships are underestimated) and corrections are needed.

One way to create a ${\mathbf{G}}^{{\text{X}}}$ matrix that, by default, is compatible with ${\mathbf{S}}$ is to use the metafounders theory [21], using $p = 0.5$ to construct ${\mathbf{G}}^{{\text{X}}}$ and using a pedigree-relationship matrix constructed with metafounders, ${\mathbf{S}}^{\gamma }$ (rules and code for this matrix and its inverse are in Additional file 1). In this method, because allele frequencies are set to 0.5, the diagonal values of ${\mathbf{G}}^{{\text{X}}}$ for males are 0.5 by construction. Note that, in the application realized on real data, we will not use that approach and will use estimated allele frequencies.

Now we consider the rank of ${\mathbf{G}}^{{\text{X}}}$. The rank of ${\mathbf{G}}^{X} = \frac{{{\mathbf{Z}}^{{\text{X}}} {\mathbf{Z}}^{{{\text{X}^{\prime}}}} }}{{2\sum {p_{i} q_{i} } }}$ is the row rank of ${\mathbf{Z}}^{{\text{X}}}$, in other words, the number of linearly independent rows. For instance, a male that receives its X-chromosome from its maternal grandsire without recombination (in its dam) results in a rank reduction of 1. Because X-chromosomes are passed on from males to offspring without recombination, and males only have one copy, this results in less “shuffling” of loci across the chromosome and therefore in higher linkage disequilibrium (LD). Thus, the row rank of ${\mathbf{Z}}^{{\text{X}}}$ and the rank of ${\mathbf{G}}^{{\text{X}}}$ are likely lower than the row rank of an autosomal chromosome of the same size, such as, for instance, bovine chromosome 2. This will be numerically evaluated later in this work.

### Treatment of dosage compensation and sex heterogeneity in traits expressed in both sexes

Many traits in livestock are expressed only in one sex (e.g., milk production), but some (e.g., growth) are expressed in both sexes. However, as for autosomes, the genetic correlation between sexes is not necessarily 1 [22]. Dosage compensation is a mechanism that balances gene expression differences in X-linked genes between sexes, e.g., [5]. This can be accomplished by randomly silencing one of the copies in females on the X-chromosome, often referred to as X-chromosome inactivation [5]. However, apart from X-inactivation, other mechanisms exist that achieve dosage compensation such as a two-fold increase of expression in males or halving both copies in females [see review by 8]. This phenomenon might explain why in spite of carrying only one copy of the X-chromosome, males present as much genetic variation in their phenotypes associated with variants from the X-chromosome as females (see for instance in [23]). Yang et al. [18] and Su et al. [7] presented three models that differ on the assumption of dosage compensation (none, full compensation (same genotypic mean), and same genotypic variance in males and females). This allows construction of a GRM for the X-chromosome, but at the price of assuming a certain factor $k$ of dosage compensation that needs to be known or assumed. Below, we present a general alternative strategy, including explicit estimation of dosage compensation. To summarize, for a trait expressed in both sexes, we propose a multiple-trait model (phenotypes in both sexes are considered as two different traits). Ideally, this bivariate approach should be applied to model genetic effects that are associated with the autosomes too, although here we focus on genetic effects associated with the X-chromosome. If the genetic correlation across sexes is 1, then the dosage compensation is a function of the covariances, ${\mathbf{G}}^{{\text{X}}}$ can be explicitly built including dosage compensation, and a single-trait model is possible as described below.

#### SNP-BLUP with variable dosage compensation

Considering (random) marker effects ${{\varvec{\upalpha}}}$, that are expressed in terms of effects in the female population, then the genotypic value ($g$) of own performance of males is ${\mathbf{g}}_{males} = k{\mathbf{M}}^{{\text{X}}} {{\varvec{\upalpha}}}$ and that of own performance of females ${\mathbf{g}}_{females} = {\mathbf{M}}^{{\text{X}}} {{\varvec{\upalpha}}}$, where $k$ allows for effects to scale differently between sexes as a result of dosage compensation for instance.

In other words, scalar $k$ considers the dosage compensation and is not bounded to predefined values. For instance, the three hypotheses of Yang et al. [18] would be modelled as: no dosage compensation ($k = 1$), full dosage compensation $(k = 2$), which results in the same mean but a doubling of the genotypic variance in males compared to females, and the same genotypic variance ($k = \sqrt 2$) (but a different mean). However, other values of $k$ are possible or even likely [8].

In practice, both ${{\varvec{\upalpha}}}$ and $k$ need to be estimated from data. For this, we propose an equivalent multiple-trait based model where $k$ becomes a covariance component, as follows:

$$\left( {\begin{array}{*{20}c} {{\mathbf{y}}_{males} } \\ {{\mathbf{y}}_{females} } \\ \end{array} } \right) = \cdots \left( {\begin{array}{*{20}c} {{\mathbf{g}}_{males} } \\ {{\mathbf{g}}_{females} } \\ \end{array} } \right) + \left( {\begin{array}{*{20}c} {{\varvec{e}}_{males} } \\ {{\varvec{e}}_{females} } \\ \end{array} } \right) =\cdots + \left( {\begin{array}{*{20}c} {{\mathbf{M}}_{males}^{{\text{X}}} } \\ 0 \\ \end{array} } \right){{\varvec{\upalpha}}}^{{\left( {males} \right)}} + \left( {\begin{array}{*{20}c} 0 \\ {{\mathbf{M}}_{females}^{{\text{X}}} } \\ \end{array} } \right){{\varvec{\upalpha}}} + \left( {\begin{array}{*{20}c} {{\mathbf{e}}_{males} } \\ {{\varvec{e}}_{females} } \\ \end{array} } \right),$$

where we keep ${{\varvec{\upalpha}}}$ for females, with the associated distribution:

$$Var\left( {\begin{array}{*{20}c} {{{\varvec{\upalpha}}}^{{\left( {males} \right)}} } \\ {{\varvec{\upalpha}}} \\ \end{array} } \right) = \left( {\begin{array}{*{20}c} {\sigma_{\alpha - m}^{2} } & {\sigma_{\alpha - m,f} } \\ {\sigma_{\alpha - m,f} } & {\sigma_{\alpha - f}^{2} } \\ \end{array} } \right) \otimes {\mathbf{I}}.$$

This model considers dosage compensation but also different effects of the same alleles in males and females, i.e. genetic correlation different from 1, e.g. [22]. In this model, variance components might be estimated, i.e. by REML. If the correlation of ${{\varvec{\upalpha}}}$ effects across phenotypes of males and females is 1, then $$\left( {\begin{array}{*{20}c} {\sigma_{\alpha - m}^{2} } & {\sigma_{\alpha - m,f} } \\ {\sigma_{\alpha - m,f} } & {\sigma_{\alpha - f}^{2} } \\ \end{array} } \right) = \left( {\begin{array}{*{20}c} {k^{2} \sigma_{\alpha }^{2} } & {k\sigma_{\alpha }^{2} } \\ {k\sigma_{\alpha }^{2} } & {\sigma_{\alpha }^{2} } \\ \end{array} } \right)$$. Thus, it is possible to check simultaneously if the genetic architecture is the same and (if it is the same, the correlation is 1) the extent of dosage compensation. Obviously, the same two-trait model (one per sex) should be run simultaneously for the autosomes and the X-chromosome to check the heterogeneity of the trait across sexes. If the genetic correlation is already known to be 1 (or if it is assumed to be 1), $k$ might be estimated with a more parsimonious univariate model accounting for heterogeneous variance.

#### Genomic BLUP for the X-chromosome with variable dosage compensation

Let us now consider genetic evaluation in a GBLUP form for own phenotype. As before, to take dosage compensation into account, we can consider a multiple-trait model equivalent to the SNP-BLUP presented before:

$$\left( {\begin{array}{*{20}c} {{\mathbf{y}}_{males} } \\ {{\mathbf{y}}_{females} } \\ \end{array} } \right) = \cdots + \left( {\begin{array}{*{20}c} {{\mathbf{u}}_{males} } \\ 0 \\ \end{array} } \right) + \left( {\begin{array}{*{20}c} 0 \\ {{\mathbf{u}}_{females} } \\ \end{array} } \right) + {\mathbf{e}},$$

with

$$Var\left( {\begin{array}{*{20}c} {{\mathbf{u}}_{males} } \\ {{\mathbf{u}}_{females} } \\ \end{array} } \right) = \left( {\begin{array}{*{20}c} {\sigma_{u - m}^{2} } & {\sigma_{u - m,f} } \\ {\sigma_{u - m,f} } & {\sigma_{u - f}^{2} } \\ \end{array} } \right) \otimes {\mathbf{G}}^{{\text{X}}}.$$

Again, the analysis should model multiple traits for both autosomes and the X-chromosome. The respective genetic variances (assuming Hardy–Weinberg equilibrium) are $\sigma_{u - males}^{2} = k^{2} \sum p_{i} q_{i} \left( {\alpha_{i}^{{\left( {males} \right)}} } \right)^{2}$ and $\sigma_{u - females}^{2} = 2\sum p_{i} q_{i} \alpha_{i}^{2}$.

If the genetic correlation across sexes is 1, we have $$\left( {\begin{array}{*{20}c} {\sigma_{u - males}^{2} } & {\sigma_{u - m,f} } \\ {\sigma_{u - m,f} } & {\sigma_{u}^{2} } \\ \end{array} } \right) = \left( {\begin{array}{*{20}c} {\frac{{k^{2} }}{2}\sigma_{u}^{2} } & {\frac{k}{\sqrt 2 }\sigma_{u}^{2} } \\ {\frac{k}{\sqrt 2 }\sigma_{u}^{2} } & {\sigma_{u}^{2} } \\ \end{array} } \right)$$ from which $k$ can be solved. The factor $\frac{{k^{2} }}{2}$ is explained because males have half the number of copies compared to females but effects are scaled by $k$. It is then possible to define a new ${\mathbf{G}}^{{\mathbf{X}}}$ explicitly accounting for the level of dosage compensation, and that can be used in single-trait analyses:

$${\mathbf{G}}^{{\text{X}}} = \frac{1}{{2\sum p_{i} q_{i} }}\left( {\begin{array}{*{20}c} {k{\mathbf{M}}_{males}^{{\text{X}}} - k1{\mathbf{p^{\prime}}}} \\ {{\mathbf{M}}_{females}^{{\text{X}}} - 2{\mathbf{p^{\prime}}}} \\ \end{array} } \right)\left( {\begin{array}{*{20}c} {k{\mathbf{M}}_{males}^{{\text{X}}} - k1{\mathbf{p^{\prime}}}} \\ {{\mathbf{M}}_{females}^{{\text{X}}} - 2{\mathbf{p^{\prime}}}} \\ \end{array} } \right)^{^{\prime}}.$$

The different matrices in Yang et al. [18] are particular cases of this GRM, setting $k = 1$, $\sqrt 2$ or $2$. As for the SNP-BLUP, more parsimonious univariate models are possible to account for heterogeneity of variances across sexes when the genetic correlation is known to be 1.

As already mentioned, an equivalent GRM can be obtained when coding males as {0,2} by using appropriate scaling factors. This coding is commonly used in dairy cattle genetics [6, 7], and amounts to assuming $k$ = 2. More importantly, for sex-specific traits (observed in a single sex) such as milk and egg production, the value of $k$ (or the choice of coding) is irrelevant.

### Genomic applications combining pedigree relationships and genotypes on the X-chromosome

#### Heritability of gene content and single step

Fernando and Grossman [16] described the pedigree-based relationship matrix ${\mathbf{S}}$ (and its sparse inverse ${\mathbf{S}}^{ - 1}$) at a random locus on chromosome X. Seen as a quantitative trait, the methods that we presented fit their modelling of gene content in the males as {0,1} and in females as {0,1,2}.

There are two major applications of pedigree relationships to the use of genotypes from markers on the X-chromosome. First, consider modelling gene content $m$ as a quantitative trait [24, 25]. Variance and covariances of $m$ across individuals are described by matrix ${\mathbf{S}}$:

$$E\left( {\begin{array}{*{20}c} {{\mathbf{m}}_{males} } \\ {{\mathbf{m}}_{females} } \\ \end{array} } \right) = \left( {\begin{array}{*{20}c} {1p} \\ {2p} \\ \end{array} } \right)$$; $$Var\left( {\begin{array}{*{20}c} {{\mathbf{m}}_{males} } \\ {{\mathbf{m}}_{females} } \\ \end{array} } \right) = {\mathbf{S}}2pq$$.

For instance, it is possible to estimate the heritability of gene content for quality control purposes [19].

Second, we can also predict gene content from ungenotyped individuals thanks to genotyped individuals using the pedigree-based matrix ${\mathbf{S}}$. The individuals in the pedigree file can be split into genotyped (subscript 2) and ungenotyped (subscript 1) individuals, and the $${\mathbf{S}} = \left( {\begin{array}{*{20}c} {{\mathbf{S}}_{11} } & {{\mathbf{S}}_{12} } \\ {{\mathbf{S}}_{21} } & {{\mathbf{S}}_{22} } \\ \end{array} } \right)$$ matrix partitioned accordingly. We will work with centered gene content, i.e. ${\mathbf{Z}}^{{\text{X}}} = {\mathbf{M}}^{{\text{X}}} - 1{\mathbf{p^{\prime}}}$ for males and ${\mathbf{Z}}^{{\text{X}}} = {\mathbf{M}}^{{\text{X}}} - 2{\mathbf{p^{\prime}}}$ for females. At a single locus, the linear prediction of ${\mathbf{z}}_{1}^{{\text{X}}}$ for ungenotyped individuals from observed genotypes (${\mathbf{z}}_{2}^{{\text{X}}}$) in genotyped individuals is ${\mathbf{z}}_{1}^{{{\hat{\text{X}}}}} = E\left( {{\mathbf{z}}_{1}^{{\text{X}}} |{\mathbf{z}}_{2}^{X} } \right) = {\mathbf{S}}_{12} {\mathbf{S}}_{22}^{ - 1} {\mathbf{z}}_{2}^{X}$ with associated variance (assuming multivariate normality) $var\left( {{\mathbf{z}}_{1}^{{\text{X}}} |{\mathbf{z}}_{2}^{{\text{X}}} } \right) = \left( {{\mathbf{S}}_{11} - {\mathbf{S}}_{12} {\mathbf{S}}_{22}^{ - 1} {\mathbf{S}}_{21} } \right)2pq$. This allows the construction of the SSGBLUP relationship matrix ${\mathbf{H}}^{{\text{X}}}$ including the X-chromosome as described next. The relationship matrix is built as a cross-product of estimated and observed ${\mathbf{Z}}^{{\text{X}}}$, considering the error in the estimation (see Christensen and Lund [3] for the details), which yields a SSGBLUP type matrix:

$${\mathbf{H}}^{{\text{X}}} = \left( {\begin{array}{*{20}c} {{\mathbf{S}}_{11} - {\mathbf{S}}_{12} {\mathbf{S}}_{22}^{ - 1} {\mathbf{S}}_{21} + {\mathbf{S}}_{12} {\mathbf{S}}_{22}^{ - 1} {\mathbf{G}}^{{\text{X}}} {\mathbf{S}}_{22}^{ - 1} {\mathbf{S}}_{21} } & {{\mathbf{S}}_{12} {\mathbf{S}}_{22}^{ - 1} {\mathbf{G}}^{{\text{X}}} } \\ {{\mathbf{G}}^{{\text{X}}} {\mathbf{S}}_{22}^{ - 1} {\mathbf{S}}_{21} } & {{\mathbf{G}}^{{\text{X}}} } \\ \end{array} } \right),$$

with the inverse, assuming invertible ${\mathbf{G}}_{{\text{X}}}^{ - 1}$:

$$\left( {{\mathbf{H}}^{{\text{X}}} } \right)^{ - 1} = {\mathbf{S}}^{ - 1} + \left( {\begin{array}{*{20}c} 0 & 0 \\ 0 & {{\mathbf{G}}_{{\text{X}}}^{ - 1} - {\mathbf{S}}_{22}^{ - 1} } \\ \end{array} } \right).$$

It is also possible to develop a so-called single step SNP-BLUP [26] to work directly with effects of markers on the X-chromosome instead of additive genotypic values.

The development above needs base allele frequencies to construct ${\mathbf{Z}}_{2}^{{\text{X}}}$ and fit ${\mathbf{H}}^{{\text{X}}}$ to the pedigree base. If these are not available, an option is to analytically integrate (unknown) base allele frequencies [27], which in practice means to use $p = 0.5$ across all loci for the construction of ${\mathbf{G}}^{{\text{X}}}$ and use the metafounder’s theory to construct ${\mathbf{S}}$. This is described in Additional file 1.

Note that if a different coding of gene content is applied, similar applications can be performed by rescaling ${\mathbf{S}}$. Indeed, if {0,1,2} coding is chosen for females and any coding {0,*k*} is chosen for males to construct a particular ${\mathbf{G}}^{{\text{X}}}$, then ${\mathbf{S}}$ can be rescaled appropriately by multiplying the (male, male) part by $k^{2}$ and the (male, female) and (female, male) parts by $k$. Rescaling **S** might be more convenient than recoding genotypes in already existing databases.

### Note on combining pedigree relationships and genotypes for markers in the pseudo-autosomal region

The PAR that behaves differently than the X-chromosome specific part represents a much smaller region (approximately 5 Mb) and is hence less likely to have an important contribution to genetic variation. The PAR has also much larger recombination rate in males, e.g. [28–30]. The rules to estimate the GRM are the same as for the autosomes and the SNPs from the PAR do not need specific rules. However, the expected relationships are not the same as for the autosomes. Indeed, there is a so-called sex-gradient in the PAR [28] because sires transmit more often their paternal haplotype (associated with the Y-chromosome) to their sons and their maternal haplotype (associated with the X-specific part) to their daughters. This probability is equal to ($1{-}r$), where $r$ is the genetic distance to the pseudo-autosomal boundary (PAB) in males. Therefore, the allelic effects (or eventually the gene content) of son $mo$ and daughter $fo$ from sire $s$ are:

$$v_{mo}^{p} = \left( {1 - r} \right)v_{s}^{p} + rv_{s}^{m} + \varepsilon_{mo}^{p},$$

$$v_{fo}^{p} = \left( {1 - r} \right)v_{s}^{p} + rv_{s}^{m} + \varepsilon_{fo}^{p},$$

where superscript $p$ and $m$ indicates paternal and maternal alleles, subscripts $mo$ and $fo$ indicate male and female offspring, subscript $s$ refers to the sire and $\varepsilon$ represents residual effects (due to sampling). These equations are similar to those that describe the transmission of a QTL from parent to offspring conditionally on a set of markers proposed by Fernando and Grossman [31]. In that case, the probability of inheritance of a paternal and maternal allele is estimated based on the markers. Here, the sex of the offspring plays the role of a marker located at the PAB. Note that for dams, there is no such gradient and transmission has equal probability as on autosomes:

$$v_{mo}^{m} = 0.5v_{d}^{p} + 0.5v_{d}^{m} + \varepsilon_{mo}^{m},$$

$$v_{{fo}}^{m} = 0.5v_{d}^{p} + 0.5v_{d}^{m} + \varepsilon _{{fo}}^{m} ,$$

where subscript $d$ stands for dam. These equations correspond to standard pedigree-based expectations. The expected relationship matrix is different for each marker on the PAR as it depends on its position. It can be estimated using the genetic distance from the PAB (measured in males), the above equations and the rules described in Fernando and Grossman [31]. Hereafter this matrix will be noted ${\mathbf{P}}^{r}$ where $r$ is the distance (in cM). Rules were also provided to compute directly the inverse of this relationship matrix [31].

## Application

### Empirical comparison of pedigree-based and genomic relationships for markers on the X-chromosome

#### Data

The dataset used in our study consisted in a sample of 6085 French Holstein individuals genotyped with the BovineSNP50 or the BovineHD genotyping arrays (Illumina, San Diego, CA). These 637 sires and 5448 dams corresponded to the French Holstein parents that have a phenotype for global recombination rate in the study by Kadri et al. [32]. For the autosomes, we conserved the 30,127 markers selected by Kadri et al. [32] after discarding monomorphic markers or those with a low call rate (lower than 0.95), markers that deviated from Hardy–Weinberg proportions, that had more than 10 Mendelian inconsistences or were associated with putative map errors. Similar filtering rules were applied to a set of 853 SNPs mapping to the X-chromosome in the ARS-UCD1.2 bovine assembly and common to the two genotyping arrays (see also [30]). The X-specific part ended at the PAB, set at position 133,300,518 [9]. X-specific markers with an average homozygosity lower than 0.98 in males were also filtered out, leaving 744 SNPs on the X-specific part and 73 SNPs on the PAR. Remaining Mendelian inconsistencies were subsequently erased. The pedigree including available ancestors contained 16,669 individuals; the oldest ancestors from each genotyped individual traced back to 7 to 16 generations in males and 9 to 17 generations in females. These genotyped males and females had more than, respectively, 90 and 95% known ancestors in the fourth pedigree generation.

We used LINKPHASE3 [33] to reconstruct haplotypes of genotyped animals, to estimate the probability of transmission of parental haplotypes to offspring at each marker position and to obtain the number of cross-overs on the X-specific part in female meiosis.

#### Comparison of genetic relationship matrices

Pedigree-based relationship matrices were estimated with our own code. For the autosomes (${\mathbf{A}}$), we used the tabular method [34] whereas for the sex-chromosome (${\mathbf{S}}$) we used the rules described in Fernando and Grossman [16]. The genomic relationship matrices (GRM) for autosomes (${\mathbf{G}}$) and the sex-chromosome (${\mathbf{G}}^{{\text{X}}}$) were computed with GCTA [18] using the first model proposed by VanRaden [20] and assuming equal variance of SNP effects. More specifically, for the X-chromosome (X-GRM), we used the coding {0,1} for males and {0,1,2} for females and then the cross-product ${\mathbf{G}}^{{\text{X}}} = \frac{{{\mathbf{Z}}^{{\text{X}}} {\mathbf{Z}}^{{{\text{X}^{\prime}}}} }}{{2\sum {\text{pq}}}}$ as described in theory and using the allele frequencies estimated in the sample, which underestimates a little the relationships compared to pedigree-based relationships. If this relationship matrix is used in a single-trait GBLUP analysis (for instance to analyze growth), multiple-trait analyses should be used to consider dosage compensation as presented in ‘Theory’ section. We estimated one GRM for all autosomes jointly and one for chromosome 2 (${\mathbf{G}}^{{{\text{BTA}}2}}$), with a physical length similar to the X-chromosome.

We started by comparing estimated relationships for different pairs of individuals (e.g., sire-son, full-sisters, paternal half-brothers, etc.). We also rescaled expected relationships in terms of correlations between animals [35]. This amounts to dividing relationships between $i$ and $j$ by the square root of the product from the diagonal elements $i$ and $j$ (on autosomes, this correlation between genetic effects is equal to the additive relationship in absence of inbreeding). This rescaling makes the relationships less dependent to variation in diagonal elements and ensures that the values are between -1 and 1, making them easier to interpret. Subsequently, we estimated the correlations between pedigree-based and genomic relationships for all elements or for off-diagonal elements only. Finally, we compared the dimensionality of the different relationship matrices by performing a singular value decomposition (SVD). First, we estimated the percentage of the overall variance explained by the $i$th pair of SVD vectors (called the $i$th SVD mode) as:

$$\frac{{sv_{i}^{2} }}{{\sum sv_{j}^{2} }}$$

where $sv_{j}$ are the singular values. Subsequently, we estimated the percentage variance captured by the $k$ largest singular values and determined the values of $k$ needed to capture 90, 95 or 99% of variance.

#### Heritability of gene content

Forneris et al. [19] proposed to estimate the heritability of gene content to perform quality control of genotypes. Here, we estimated heritability of gene content for the 744 markers on the X-chromosome (X-specific part) by using either the autosomal additive relationship matrix ${\mathbf{A}}$, or the X-chromosome relationship matrix ${\mathbf{S}}$. In both cases, the relationship matrices were computed from pedigree data. The gene content was equal to the number of copies of the reference alleles (ranging from 0 to 2 in females and 0 to 1 in males). Variance components were estimated with the AI-REML algorithm implemented in the blupf90 package [36]. Comparisons allowed to check which relationship matrix (${\mathbf{S}}$ or ${\mathbf{A}}$) best fits the data, and at the same time, whether covariances of gene content at a locus are correctly described by ${\mathbf{S}}$ as expected (i.e., if the heritability estimate is 1, the theory fits the reality). First, the heritability was estimated by including all animals. Most of the individuals were females (90%) and meiosis is similar on the X-chromosome and the autosomes whereas males have a more deviant pattern. Therefore, we also worked exclusively with males to obtain more contrasted comparisons.

Finally, we also estimated heritability of gene content for markers on the PAR with a similar approach. In addition, to ${\mathbf{A}}$ and ${\mathbf{S}}$, we also estimated expected relationships at different distances from the PAB (0.1, 5, 10, 20, 30, 40 and 50 cM) by combining the transmission probabilities described in Methods and the rules from Fernando and Grossman [31] to estimate ${\mathbf{P}}^{r}$.

## Results

### Expected relationships for markers on the X-chromosome

Examples of expected relationships on the X-chromosomes and on the autosomes are in Table 1, including in terms of correlations between individuals. Contrary to autosomes for which the relationship does not depend on the sex of an individual, for the X-chromosome, parent and offspring genders matter. For instance, sire-son pairs or paternal half-brothers (with unrelated dams) have a null expected relationship. Conversely, correlations between additive genetic effects from maternal half-brothers are expected to be higher than for autosomes. Similarly, correlations between genetic effects from mother-son pairs (0.71) or from paternal half-sisters (0.50) are higher than for autosomes (0.50 and 0.25, respectively). It should also be noted that maternal half-brothers and full-brothers have the same expected relationship (0.25). More generally, we observe that expectations vary according to the sex of the full-sibs or half-sibs and according to the sex of the common parent for half-sib pairs.

**Table 1:** Expected (pedigree-based) additive genetic relationships on the autosomes and on the X-chromosome (specific part) for different categories of animals

Relationship class	Autosomes		X-chromosome	
	Relationships	Correlations	Relationships	Correlations
Sire/son	0.50	0.50	0.00	0.00
Sire/daughter	0.50	0.50	0.50	0.71
Dam/son	0.50	0.50	0.50	0.71
Dam/daughter	0.50	0.50	0.50	0.50
Paternal half-sibs (two males)	0.25	0.25	0.00	0.00
Paternal half-sibs (male/female)	0.25	0.25	0.00	0.00
Paternal half-sibs (two females)	0.25	0.25	0.50	0.50
Maternal half-sibs (two males)	0.25	0.25	0.25	0.50
Maternal half-sibs (male/female)	0.25	0.25	0.25	0.35
Maternal half-sibs (two females)	0.25	0.25	0.25	0.25
Full-sibs (two males)	0.50	0.50	0.25	0.50
Full-sibs (male/female)	0.50	0.50	0.25	0.35
Full-sibs (two females)	0.50	0.50	0.75	0.75
Diagonal elements (males)	1.00	1.00	0.50	1.00
Diagonal elements (females)	1.00	1.00	1.00	1.00

The relationships are also represented in terms of correlations (i.e., dividing by the square root of 0.5 for each male involved in the relationship)

### Pedigree-based and realized relationships in the French Holstein cattle data

**Fig. 1:**
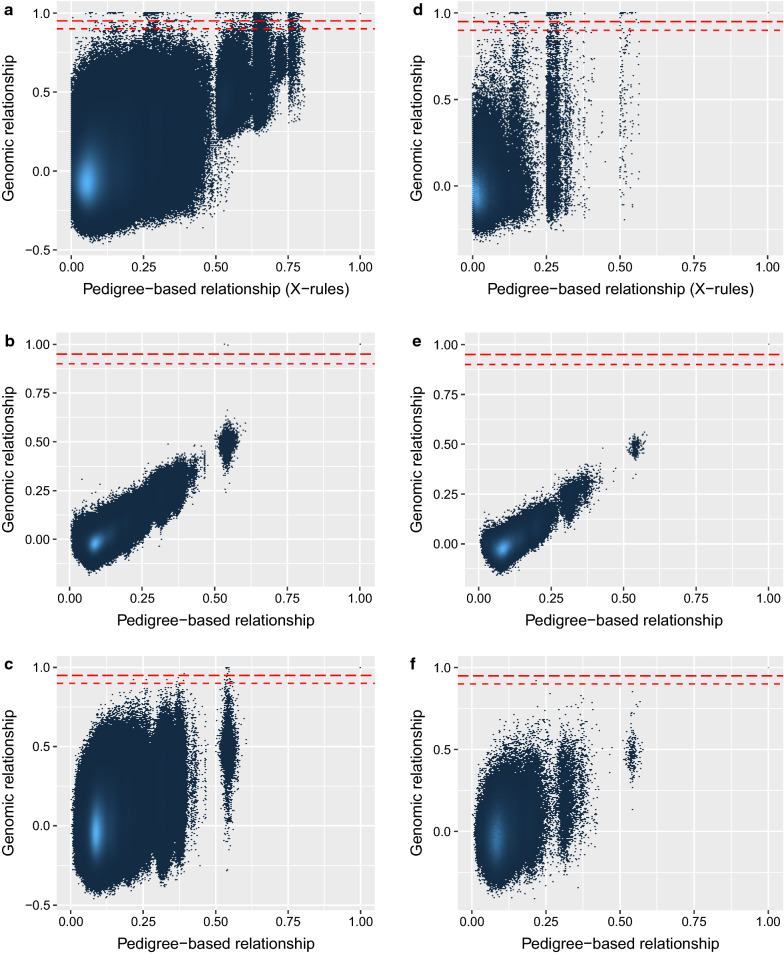
Comparison of expected and realized correlations between additive genetic effects estimated on chromosome-X (**a**, **d**), whole-genome (**b**, **e**) and for the chromosome 2 (**c**, **f**). Right panels (**d**–**f**) were obtained using relationships among males only. A hexbin function was used with a 200 $\times$ 200 grid. The color scale indicates the number of relationships having a given value

Expected (pedigree-based) and realized (marker-based) additive genetic relationships between the 6085 French Holstein individuals were estimated. The estimated correlations between polygenic effects for selected categories of individuals are in Table 2, for the X-chromosome, for the 29 autosomes jointly, and for chromosome 2. We observe that the genomic relationships obtained with genetic markers fit the pedigree-based expectations (genomic relationships are a bit lower because observed, not base, allele frequencies were used), although with some variability. The realized genomic relationships were clearly closer to the expected relationships derived for the X-chromosome with the rules from Fernando and Grossman [16] than to the expectations derived for the autosomes. The opposite was observed for realized relationships on the autosomes (see Additional file 2: Tables S1, S2 and S3 for more details on the distributions). To quantify these observations, we computed the correlations between realized and expected relationships. These were equal to 0.52, 0.81 and 0.33 for the X-chromosome, the whole-genome (the 29 autosomes) or chromosome 2, respectively. When using only the off-diagonal elements, these correlations were respectively 0.51, 0.79 and 0.31, and 0.40, 0.79 and 0.35 when considering males only. Correlations between off-diagonal elements from ${\mathbf{S}}$ and ${\mathbf{G}}$ or from ${\mathbf{A}}$ and ${\mathbf{G}}^{{\text{X}}}$, were as expected lower (respectively, 0.63 and 0.42), which indicates that ${\mathbf{S}}$ fits ${\mathbf{G}}^{{\text{X}}}$ better and ${\mathbf{A}}$ fits ${\mathbf{G}}$ better (the correlation between elements from ${\mathbf{A}}$ and ${\mathbf{S}}$ being equal to 0.76 in our dataset). The genetic relationships for chromosome 2 presented the lowest correlations between expected and realized values whereas they were highest for relationships obtained from genome-wide markers (on all 29 autosomes). In Fig. 1, we plotted realized and expected relationships expressed as the additive genetic correlations between animals as defined by Wright [35]. We observed a high level of variation along the Y-axis for genomic relationships estimated with markers on the X-chromosome.

**Table 2:** Comparison of average realized (marker-based) and expected (pedigree-based) additive genetic relationships on the X-chromosome (specific part), on all autosomes and on chromosome 2 for different categories of animals

Relationship class	Chromosome X		All autosomes		Chromosome 2	
	Pedigree	Genomic	Pedigree	Genomic	Pedigree	Genomic
Sire/son	0.031	− 0.027	0.541	0.482	0.541	0.470
Sire/daughter	0.721	0.697	0.543	0.482	0.543	0.474
Dam/son	0.721	0.700	0.545	0.481	0.545	0.462
Dam/daughter	0.539	0.479	0.545	0.484	0.545	0.476
Paternal half-sibs (two males)	0.066	0.038	0.324	0.230	0.324	0.218
Paternal half-sibs (male/female)	0.064	− 0.021	0.324	0.225	0.324	0.207
Paternal half-sibs (two females)	0.546	0.479	0.322	0.226	0.322	0.208
Maternal half-sibs (two males)	0.522	0.523	0.318	0.229	0.318	0.175
Maternal half-sibs (male/female)	0.387	0.339	0.321	0.229	0.321	0.210
Maternal half-sibs (two females)	0.309	0.228	0.321	0.232	0.321	0.222
Full-sibs (two males)	0.510	0.416	0.544	0.476	0.544	0.510
Full-sibs (male/female)	0.383	0.315	0.546	0.477	0.546	0.446
Full-sibs (two females)	0.768	0.734	0.542	0.482	0.542	0.473
Diagonal elements (males)	1.000	1.000	1.000	1.000	1.000	1.000
Diagonal elements (females)	1.000	1.000	1.000	1.000	1.000	1.000

Relationships are expressed as correlations

**Fig. 2:**
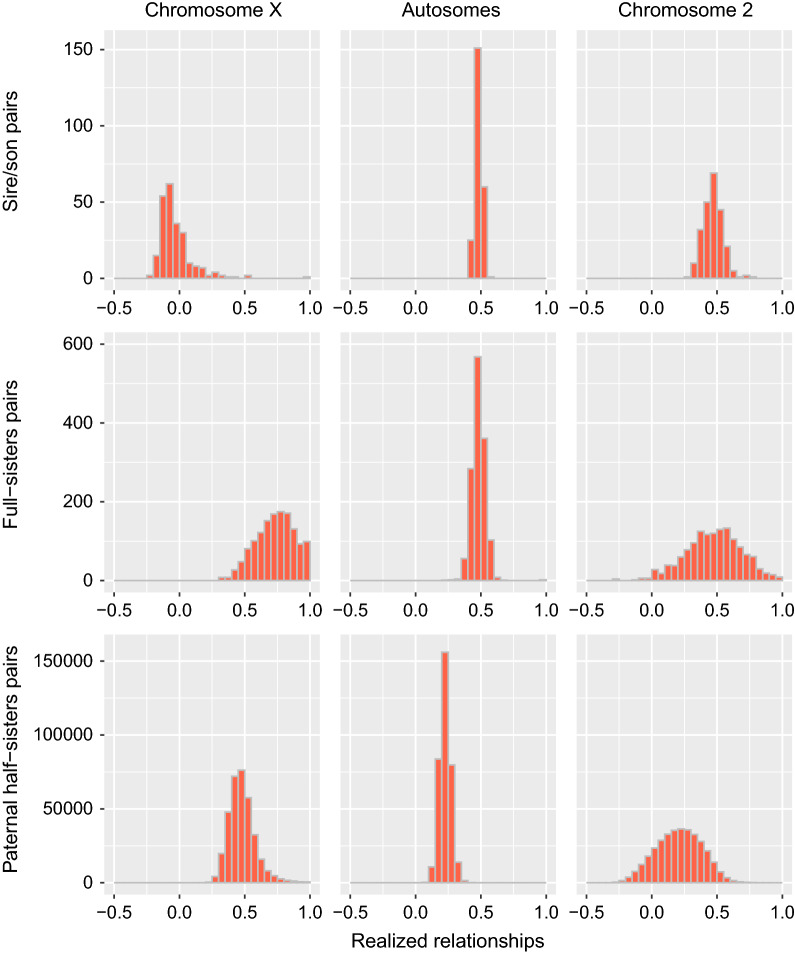
Distribution of genomic correlations estimated with markers on the X-chromosome (left panel), on the 29 autosomes (center) or on chromosome 2 (right panel). Relationships were estimated for sire/sons relationships (top), full-sisters (center) and paternal half-sisters (bottom)

Many pairs of individuals had a high realized genomic correlation (> 0.90), which indicates that their respective X-chromosomes were almost identical. This trend was observed for the whole range of expected relationships; some pairs of individuals that were expected to have a null relationship had identical X-chromosomes (see also Fig. 2). More than 1800 pairs of individuals had such high genomic correlations and the values were higher than 0.99 for more than 628 pairs (see Table 3). Such high correlations were rare when relationships were estimated on chromosome 2 (5 pairs with a correlation higher than 0.99). Finally, such a pattern is not observed on the whole-genome (only 2 identical individuals), which is expected since the relationship is estimated on a larger number of independent chromosomes (segregating separately during meiosis). Overall, the frequency of high genomic correlations were 50 to 100 times higher on chromosome X than on chromosome 2 or even more when compared to estimates computed with all the autosomes (see Table 3). The frequency of high genomic correlations on the X-chromosome was approximately 10 times higher when considering males only.

**Table 3:** Dimensionality of different genomic relationship matrices

Statistic	X-chromosome	BTA2	Autosomes
Number of positive SV	5921	5693	6085
Number of SV accounting for 90% of total	15	24	105
Number of SV accounting for 95% of total	25	39	250
Number of SV accounting for 99% of total	53	85	793
Number of SV accounting for 99.9% of total	122	191	1977
Proportion of correlations > 0.90	1.0e−4	1.7e−6	1.1e−7
Proportion of correlations > 0.95	5.4e−5	5.4e−7	1.1e−7
Proportion of correlations > 0.99	3.4e−5	2.7e−7	1.1e−7
Proportion of correlations > 0.90 (males only)	1.6e−3	4.9e−6	0
Proportion of correlations > 0.95 (males only)	1.1e−3	0	0
Proportion of correlations > 0.99 (males only)	8.9e−4	0	0

The dimensionality was assessed based on the singular value decomposition (SVD). The frequency of high genomic correlations between individuals are also indicated (either in the entire genotyped samples, either in genotyped males only)

Several factors explain the large number of high genomic correlations between pairs of individuals on the X-chromosome. First, males have only one copy of the X-chromosome and require thus only one pair of chromosomes to be identical, whereas on chromosome 2, two pairs of chromosomes need to be simultaneously identical. In addition, chromosomes are transmitted without recombination from sires to daughters. On average, in our phased dataset dams transmitted non-recombining X-chromosomes in 26.4% of their gametes (see also [30]). As a result, two maternal half-brothers have a $\sim 0.5 \times 0.264^{2}$ probability of inheriting the same chromosome (1/32). Similarly, a son and its maternal grand-sire have a 0.5 $\times$ 0.264 probability of inheriting the same chromosome (representing 1/8 of such pairs). Even females have sometimes an increased probability of inheriting two IBD chromosomes. Two full-sisters will automatically inherit the same paternal chromosome and have a $0.5 \times 0.264^{2}$ probability of inheriting the same maternal chromosome, e.g., resulting in the same probability (1/32) than for two maternal half-brothers.

Genomic relationships for the X-chromosome seemed visually more variable than estimates for chromosome 2 (Fig. 1), although genetic correlations between expected and realized values were higher for the X-chromosome. These higher correlations might be because expected values are spread across a broader range on the X-chromosome (for instance, some relationship is expected at 0.75). Finally, to illustrate further the distribution of the relationships obtained with different chromosomes, in Fig. 2, we plotted the distribution of genomic correlations for three categories of individuals: sire and sons (representing the selected individuals with a high contribution to genetic progress), full-sisters, and paternal half-sisters (producing cows). In the three cases, average realized relations were, as expected, different for the X-chromosome and were much less variable when using all the autosomes. We also observed a huge variation for sire/sons relationships on the X-chromosome with values equal to 1 for some pairs although the mean value was close to 0. In full-sisters and half-sisters, the genomic correlations seemed more variable when they were estimated for chromosome 2. Standard deviations were larger for ${\mathbf{G}}^{{\text{X}}}$ for relationships between two males and smaller for relationships between females (see Additional file 2 Table S1, S2 and S3).

### Dimensionality of genomic relationship matrices

Because of this different behavior, the GRM for the X-chromosome ${\mathbf{G}}^{{\text{X}}}$ has a reduced dimensionality compared to the whole-genome GRM or to ${\mathbf{G}}^{{{\text{BTA}}2}}$ (Table 3). For instance, the number of singular values needed to capture 99% of the total GRM variance was equal to 53 for the X-chromosome, 85 for chromosome 2, and 793 for the autosomes. Thus, ${\mathbf{G}}$ has a higher dimensionality (roughly 10$\times$) than ${\mathbf{G}}^{{{\text{BTA}}2}}$, which has a higher dimensionality (roughly 1.5$\times$ larger, in spite of being a chromosome of the same physical length) than ${\mathbf{G}}^{{\text{X}}}$. The number of non-zero singular values was equal to 5921, 5693 and 6085 for the X-chromosome, chromosome 2 and the autosomal GRM. Consequently, both GRM obtained for a single chromosome were non-positive definite.

### Heritability of gene content for markers on the X-chromosome (PAR included)

**Fig. 3:**
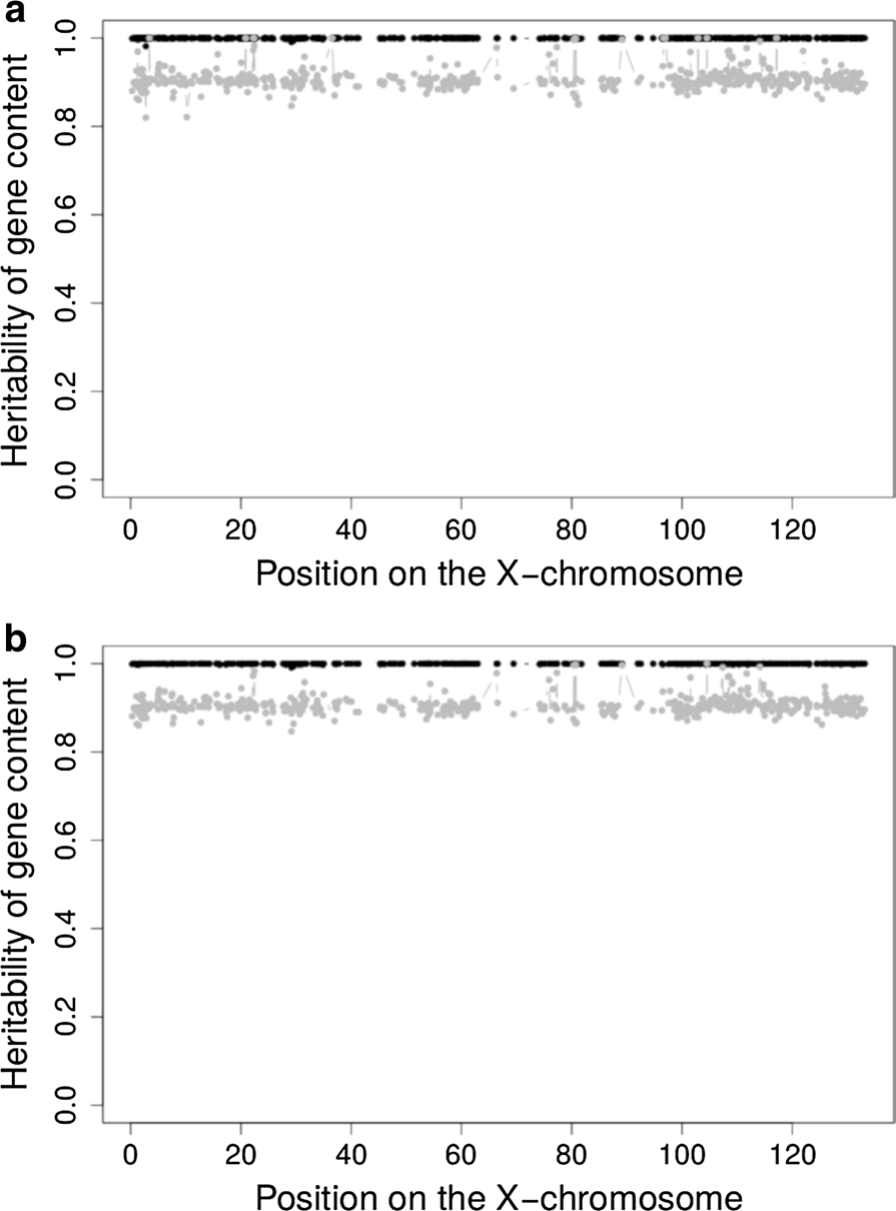
Heritability of the gene content along the X-chromosome (X-specific part). **a** For all genotyped individuals. **b** For genotyped males only. Black and gray dots indicate heritabilities estimated with the pedigree-based relationships using rules specific to the X-chromosome (${\mathbf{S}}$) and general rules for the autosomes (${\mathbf{A}}$), respectively

When the heritability of gene content for markers on the X-chromosome (X-specific part only) was estimated on all animals (males and females, simultaneously), the values estimated with ${\mathbf{S}}$ were on average equal to 0.9997 and higher than 0.95 for the 543 selected SNPs with a minor allele frequency higher than 0.05 (Fig. 3). When using ${\mathbf{A}}$, the average estimated heritability dropped to 0.9045 and was systematically lower than the estimates obtained with ${\mathbf{S}}$ (from -0.0019 to -0.1791 and -0.0952 on average). Thus, the relationship matrix constructed using the rules for the sex-chromosome [16] had a perfect fit with the “natural” gene contents for markers on the X-chromosome. When the gene content was estimated on males only, estimated heritabilities were lower and differences were more contrasted. More variation was expected since fewer individuals were used (approximately 10%). With the rules derived for the X-chromosome, 49 (155) estimates were lower than 0.95 (0.99) and the average was 0.985. Using ${\mathbf{A}}$, heritability estimates deteriorated, with 541 SNPs that had a value lower than 0.95. In fact, only two SNPs with a MAF equal to 0.05 had a value higher than 0.95. The heritabilities obtained with ${\mathbf{S}}$ were always higher (Fig. 3). Overall, the high heritabilities obtained when using all the individuals indicate that pedigree-based relationships in ${\mathbf{S}}$ describe properly the covariance of gene content across individuals, and therefore the expected (${\mathbf{S}}$) and observed (${\mathbf{G}}^{{\text{X}}}$) relationship matrices can be combined in a unique matrix, for instance in the scope of a SSGBLUP. When gene dosage was coded as {0,2} in males (instead of {0,1} as previously), the estimated heritabilities with ${\mathbf{S}}$ dropped as expected whereas those obtained with ${\mathbf{A}}$ increased (see Additional file 3 Figure S1). However, when only males were considered, ${\mathbf{A}}$ performed poorly again indicating that this matrix badly describes gene content on the X-chromosome, independently of coding strategy in males. Importantly, appropriate rescaling of ${\mathbf{S}}$ (multiplying elements by 2 for each male involved in the relationship) resulted in an average heritability of 0.9968 across SNPs.

**Fig. 4:**
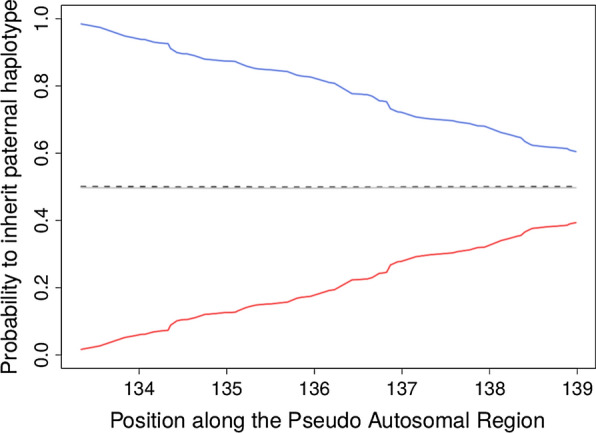
Illustration of the sex-gradient for markers in the pseudo-autosomal region (PAR). The probability for offspring to inherit the paternal haplotype from their parent was estimated with LINKPHASE3. These probabilities are plotted for transmission from sires to son (blue) and daughters (red) and from dams to sons (dashed black) and daughters (gray)

**Fig. 5:**
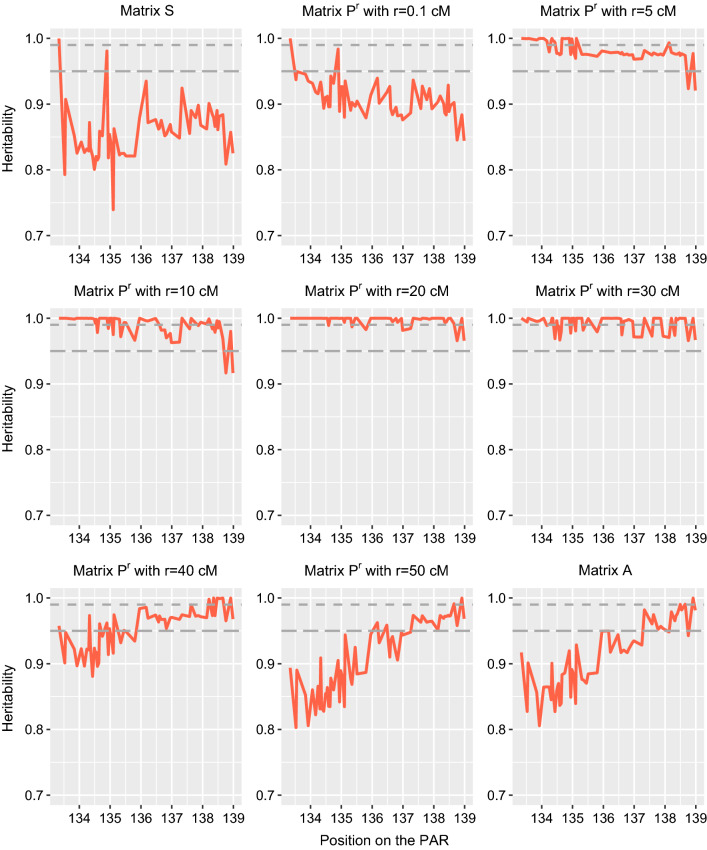
Heritability of the gene content for markers in the pseudo-autosomal region (PAR). Heritabilities were estimated with the different expected relationship matrices including ${\mathbf{S}}$ (sex-specific rules), ${\mathbf{A}}$ (autosomes) and expected relationships for markers from the PAR located at $r$ cM form the pseudo-autosomal boundary (PAB)

For markers on the PAR (60 markers with a MAF higher than 0.05), we clearly observed that the probability for sires to transmit their paternal or maternal haplotypes is a function of the sex of their offspring and the genetic distance from the PAB (Fig. 4). At the PAB, sons (daughters) inherited systematically the paternal (maternal) haplotype from the sires. At the tip of the chromosome, the disequilibrium was less strong but still present. Conversely, dams transmitted with equal probability their two haplotypes to both sons and daughters. As expected, heritability of gene content estimated with ${\mathbf{A}}$ or ${\mathbf{S}}$ was low along the whole PAR (Fig. 5). ${\mathbf{S}}$ performed best at the PAB and ${\mathbf{A}}$ at the other end of the PAR. When expectations were estimated using the sex of the offspring as a marker and the genetic distance to the PAB (see Methods), we clearly observed that relationships matrices estimated with a short genetic distance fitted well the markers that were close to the PAB and poorly markers at the end of the chromosome (Fig. 5). The opposite was true for relationships estimated with long genetic distances from the PAB. Both relationships matrices behaved imperfectly for markers located in the middle of the PAR. In that case, the use of intermediate genetic distances worked well. This indicates that for markers on the PAR, the expected relationships vary according to the distance to the PAR as we predicted. The optimal relationship matrix on the PAR could be the average of all these matrices (one matrix estimated for each SNP position). An alternative would be to use a matrix ${\mathbf{P}}^{r}$ at a moderate distance (e.g., 20 cM) since it performed relatively well for the entire PAR (Fig. 5). Our results also suggest that the heritability of gene content could be used to estimate the genetic distances on the PAR, although other methods already exist for that purpose e.g. [37].

### Combining ${\mathbf{G}}^{{\text{X}}}$ and ${\mathbf{S}}$ in a single matrix

In our data, ${\mathbf{G}}^{{\text{X}}}$ was non-positive definite. One strategy to bend ${\mathbf{G}}^{{\text{X}}}$ would be to combine it with ${\mathbf{S}}$. As an example, we used a linear regression to scale the values of ${\mathbf{G}}^{{\text{X}}}$ such that both the average of the diagonal elements and the average from all the elements are identical to the corresponding values from ${\mathbf{S}}$ [38]. To that end, first, we equalized variances in males and females (e.g., multiplying the male variance by 2). The value of the regression coefficient was close to 1 (0.9466) and the intercept was equal to 0.0963. Then, we obtained a combined GRM as ${\mathbf{G}}^{{\text{X*}}} = {\upalpha }{\mathbf{S}} + {\upbeta }{\mathbf{G}}^{{\text{X}}}$, which resulted positive definite. Indeed, with ${\upalpha }$ equal to 0.05 or 0.10, the smallest singular values were higher than 0 (respectively, 1.8e-5 and 2.0e-5).

## Discussion

The X-chromosome genetic relationship matrix (${\mathbf{G}}^{{\text{X}}}$) allows to perform genetic studies such as the estimation of its contribution to phenotypic or genetic variation. Several studies suggested that this contribution might be large for certain traits, e.g. [10–12]. Similarly, this GRM could be used to improve genomic predictions when this chromosome contributes significantly to the trait. X-specific relationships might also be useful in studies of relationships in the wild. Since expected relationships for the X-chromosome are different compared to those for autosomes, they provide additional information to determine relatedness between individuals. However, here, we have illustrated that the use of ${\mathbf{G}}^{{\text{X}}}$ is not trivial because individuals sharing their entire X-specific chromosome are relatively frequent since males carry only one copy and transmit it without recombination. As a result, ${\mathbf{G}}^{{\text{X}}}$ might be regularly non-positive definite (depending on the sample) and consequently non invertible, thus causing computational problems. Overall, we observed that the X-chromosome (X-specific part) has a lower dimensionality. This is consistent with the smaller number of chromosomes in the sample and is expected since certain relationships are estimated between individuals that have a single chromosome (we observed more extreme relationships between males). In species with a balanced sex-ratio, the effective population size (N_e_) for the X-chromosome is also smaller, three quaters of the autosomal N_e_ [39]. However, when the number of males is much smaller than the number of females, N_e_ can be slightly larger for the X-chromosome than for the autosomes [15, 40]. The lower dimensionality is also related to the reduced recombination rate on the X-chromosome (male chromosomes are transmitted without recombination) resulting in less shuffling and higher LD levels, e.g. [15, 41]. More generally, the level of diversity is also lower on the X-chromosome [39].

To address numerical problems associated with reduced dimensionality, statistical methods known as “bending” can be applied to render ${\mathbf{G}}^{{\text{X}}}$ positive definite. ${\mathbf{G}}^{{\text{X}}}$ can be bended using an identity matrix but the use of ${\mathbf{S}}$ might better preserve the genetic relationships. Indeed, we illustrated that for markers on the X-specific part, ${\mathbf{S}}$ and ${\mathbf{G}}^{{\text{X}}}$ have similar expectations and, thus, can be combined. In particular, the heritability of gene content obtained with ${\mathbf{S}}$ confirmed this point. We also showed that for markers on the PAR, expectations are different and that ${\mathbf{P}}^{r}$ had similar expectations to ${\mathbf{G}}$ for these markers. Combination of ${\mathbf{G}}^{{\text{X}}}$ and ${\mathbf{S}}$ (or eventually ${\mathbf{G}}$ and ${\mathbf{P}}^{r}$ for the PAR) would not only be useful for bending purposes, it would also allow to combine genotyped and ungenotyped individuals as for the prediction of gene content for a locus with a major effect [24, 25] or in the single-step GBLUP context. This merging of genotyped and ungenotyped individuals might be useful for both genomic predictions [2, 3] and association studies [4] on the X-chromosome. In the case of such a GWAS relying on GBLUP or SSGBLUP, we recommend the use of ${\mathbf{S}}$ and ${\mathbf{G}}^{{\text{X}}}$ (or ${\mathbf{P}}^{r}$ and ${\mathbf{G}}$) rather than ${\mathbf{A}}$ or ${\mathbf{G}}$, as done in previous studies [42].

However, several algorithms can be considered to estimate ${\mathbf{G}}^{{\text{X}}}$. Here, we used an algorithm similar to the first method proposed by VanRaden [20]. We obtained similar observations when the relationships were estimated using the rules proposed by Amin et al. [43] (also known as VanRaden second method), giving more weight to rare alleles. Alternatively, a similarity matrix, e.g. [44, 45]. can be obtained using the same allele frequency for all SNPs (0.5). Interestingly, with such an approach, all the diagonal elements for males would be equal to 0.5 as for ${\mathbf{S}}$, whereas deviations, sometimes large, are observed with the two other approaches. In addition, this approach is in line with the use of metafounders as presented in the Theory section.

As previously mentioned, when a SSGBLUP is considered, good compatibility of ${\mathbf{G}}^{{\text{X}}}$ and ${\mathbf{S}}$ is required. In addition, it is likely that different origins (male population and female population, perhaps evolving over years or origins as in genetic groups) need to be modelled. A solution for both problems is the use of metafounders [21], for which ${\mathbf{G}}^{{\text{X}}}$ is simply obtained by setting allele frequencies to 0.5 and ${\mathbf{S}}$ is obtained by fitting male and female metafounders (as many pairs as the number of genetic groups considered), and rules for ${\mathbf{S}}$ and its inverse ${\mathbf{S}}^{ - 1}$ are a simple modification of Fernando and Grossman [16] as illustrated in the Julia code provided in Additional file 4.

Importantly, we observed that ${\mathbf{G}}^{{\text{X}}}$ deviates from its expectations ${\mathbf{S}}$, more than ${\mathbf{G}}$ from ${\mathbf{A}}$. Thus, ${\mathbf{S}}$ is not a perfect predictor of ${\mathbf{G}}^{{\text{X}}}$ and it is consequently important to use realized relationships for the X-chromosome as much as possible in applications including genomic predictions or genetic variance partitioning. However, as we mentioned earlier, bending techniques are required and might result in a loss of information. For these reasons, the best strategy might be a SNP-BLUP on the X-chromosome or a single-step method that does not require bending, in the spirit of Fernando et al. [26].

Certain aspects related to the use of the X-chromosome in genomic applications were not investigated in the current study. For instance, we expressed additive genetic effects either on performances of daughters, or on own performances. However, additive genetic effects can be expressed also on other scales. For instance, genetic effects transmitted by sires to their daughters are different from genetic effects transmitted to their sons. Therefore, different prediction transmitting abilities might be proposed. VanRaden et al. [6] give more details on these aspects in the context of genomic evaluation in dairy cattle. Genotypes on the X-chromosome require also specific phasing, e.g. [46, 47] or imputation strategies [47–49].

## Conclusions

The X-chromosome has often been ignored although it might have important contributions to the genetic variation of complex traits. Certain genomic applications that combine genotyped and ungenotyped individuals require the combination of pedigree-based and realized relationship matrices. For markers on the X-chromosome, specific rules have been developed for both matrices (respectively, ${\mathbf{S}}$ and ${\mathbf{G}}^{{\text{X}}}$). In our study, we proposed to estimate dosage compensation using multiple-trait models instead of assuming a predefined value. Then, we showed theoretically and empirically that both relationship matrices have the same expectations. Therefore, we recommend combining ${\mathbf{G}}^{{\text{X}}}$ with ${\mathbf{S}}$ in applications related to gene content or in SSGBLUP approaches. We also observed that realized relationships present strong levels of variation around expected values and ${\mathbf{S}}$ is hence not a perfect predictor of ${\mathbf{G}}^{{\text{X}}}$. In addition, many individuals share entire chromosomes and have relationships close to 1. Thus, for markers on the X-chromosome, a SNP-BLUP strategy might be a good strategy since it relies on the realized relationships while having less numerical problems than a GBLUP relying on the GRM.

## Supplementary information

**Additional file 1** Metafounder’s theory as applied to the S matrix, rules and code for this matrix and its inverse.

**Additional file 2: Table S1.** Comparison of realized (marker-based) and expected (pedigree-based) additive genetic relationships on the X-chromosome (specific part) for different categories of animals. **Table S2.** Comparison of realized (marker-based) and expected (pedigree-based) additive genetic relationships on the autosomes (all together) for different categories of animals. **Table S3.** Comparison of realized (marker-based) and expected (pedigree-based) additive genetic relationships on the BTA2 for different categories of animals.

**Additional file 3: Figure S1.** Heritability of the gene content along the X-chromosome (X-specific part), when males considered homozygous are coded as {0,2}. A. For all genotyped individuals, B. For genotyped males only. Black and gray dots indicate heritabilities estimated with the pedigree-based relationships using rules specific to the X-chromosome (**S**) and general rules for the autosomes (**A**), respectively.

**Additional file 4.** Julia code.

## Data Availability

The data that support the findings of this study (genotypes) belong to third parties (INRAE and Allice). Restrictions apply to the availability of these data that are not publicly available. The authors can be contacted for a reasonable request and with permission of data owners.
