# Supplementary material for: Theoretical and empirical comparisons of expected and realized relationships for the X-chromosome
Source: Genet Sel Evol. 2020 Aug 20;52:50. doi: 10.1186/s12711-020-00570-6 (PMC7441635; doi:10.1186/s12711-020-00570-6)
Supplement: Supplementary file 1 — Additional file 1 Metafounder’s theory as applied to the S matrix, rules and code for this matrix and its inverse. [file 12711_2020_570_MOESM1_ESM.pdf]

Metafounder's theory as applied to S matrix

Here we obtain relationships at individuals in the pedigree base. Assume that relationships across gametes in the base population is $\frac{\gamma}{2}$, i.e. if we draw two gametes at random they are identical with probability $\frac{\gamma}{2}$.

Consider two males. Then the *coancestry* (probability that one gene drawn of two males i and j is 1 with themselves (there is only one copy at the locus) and $\frac{\gamma}{2}$ with each other. Scaled to the scale of females' variance component (dividing by 2) this gives $S_{ii}^{\gamma} = 0.5$ and $S_{ij}^{\gamma} = \frac{\gamma}{4}$.

Now consider two females i and j. The self-coancestry of the two females is $0.5 + \frac{\gamma}{4}$ because there are two ways of sampling copies A and B:

- A and A (prob of alikeness =1)
- A and B (prob of alikeness =$\frac{\gamma}{2}$)

Multiplied by 2 this gives relationship $S_{ii}^{\gamma} = 1 + \frac{\gamma}{2}$.
Across the two females, there are four ways of sampling, each of of them with probability of alikeness $\frac{\gamma}{2}$, resulting in average coancestry $\frac{\gamma}{2}$ and average relationship $S_{ij} = \gamma$.

Last, female i and male j. There are two ways of sampling pairs of copies because male only has one copy and females has 2, each of them with probability of alikeness $\frac{\gamma}{2}$. When this is scaled to the genetic variance of females' population, this gives $S_{ij} = \frac{\gamma}{2}$

The next matrix shows the relationship matrix S^{γ} of 4 individuals (2 males and 2 females, in this order)

$$\begin{pmatrix} 0.5 & \frac{\gamma}{4} & \frac{\gamma}{2} & \frac{\gamma}{2} \\ & 0.5 & \frac{\gamma}{2} & \frac{\gamma}{2} \\ & & 1+\frac{\gamma}{2} & \gamma \\ & & & 1+\frac{\gamma}{2} \end{pmatrix}$$

The $\boldsymbol{S}^{\gamma}$ matrix can be constructed using Fernando and Grossman tabular method, starting with *two* metafounders, one a "male" metafounder and the other a "female" metafounder, having a relationship $\Gamma^{x} = \begin{pmatrix} \frac{\gamma}{4} & \frac{\gamma}{2} \\ \frac{\gamma}{2} & \gamma \end{pmatrix}$. This matrix is not full rank but a pseudo-inverse may be used. Another option is to use the matrix $\Gamma^{x} = \begin{pmatrix} \frac{\gamma_m}{4} & \frac{\gamma_{m,f}}{2} \\ \frac{\gamma_{f,m}}{2} & \gamma_f \end{pmatrix}$ which considers possibly different allele frequencies in the founder male and female population, i.e. $\gamma_m = 8Var(p^m)$, $\gamma_f = 8Var(p^f)$, $\gamma_{m,f} = 8Cov(p_m, p_f)$ are functions of the variance and covariances of allele frequencies within and across male and female populations.

To build the inverse of S^{γ}, the algorithm reduces to

1. Invert Γ^x to form the corresponding block of $S^{(\gamma)-1}$. If it is not invertible, then use the Moore-Penrose pseudo-inverse.

2. Compute inbreeding coefficients for all individuals (including metafounders) as $S_{ii} - 1$. Note that this gives -0.5 for males.

3. For each animal *i* (not for metafounders):

   a. compute the variance of mendelian sampling as:
      i. Females: $d = 1 - \frac{1}{4}(1 + F_d) - (1 - F_s)$
      ii. Males: $d = 0.5 - \frac{1}{4}(1 + F_d)$

   b. Add elements to the inverse $S^{(\gamma)-1}$:
      i. Females: add elements of $\frac{w'w}{d}$ where $w = (1/2 \quad 1 \quad -1)$ to the corresponding elements in the outer product of locations $(d \quad s \quad i)$ (i.e. the usual rules but all ancestors are known).
      ii. Males: same with $w = (1/2 \quad 0 \quad -1)$.

The proof of this method follows. Solkner and Tier (1992) showed that using partitioned matrix algebra and considering rules for incremental construction of a matrix, the inverse of a matrix can be built by contributions starting from a smaller matrix. In our case, the relationship matrix at chromosome X is obtained by the tabular method as

$$\boldsymbol{S}_i = \begin{pmatrix} \boldsymbol{S}_{i-1} & \boldsymbol{S}_{i-1}\boldsymbol{t}_i \\ \boldsymbol{t}_i'\boldsymbol{S}_{i-1} & s_{ii} \end{pmatrix}$$

For males, vector $\boldsymbol{t}_i$ contains zeros except for 0.5 in the location of the dam of i, and $s_{ii} = 0.5$. For females, $\boldsymbol{t}_i$ contains 0.5 in the location of the dam and 1 in the location of the sire, and $s_{ii} = 1 + s_{d,s}$. Partitioned matrix algebra gives

$$\boldsymbol{S}_i^{-1} = \begin{pmatrix} \boldsymbol{S}_{i-1}^{-1} & \boldsymbol{0} \\ \boldsymbol{0} & 0 \end{pmatrix} + (s_{ii} - \boldsymbol{t}_i'\boldsymbol{S}_{i-1}\boldsymbol{t}_i)^{-1} \begin{pmatrix} \boldsymbol{t}_i\boldsymbol{t}_i' & -\boldsymbol{t}_i \\ -\boldsymbol{t}_i' & 1 \end{pmatrix}$$

From here, we derive contributions to the setup of $\boldsymbol{S}^{-1}$. Skipping the 0 values of vector $\boldsymbol{t}_i$, $\boldsymbol{t}_i'\boldsymbol{S}_{i-1}\boldsymbol{t}_i$ contains only relationship across dams and sires of *i*. In the female case, this is $\begin{pmatrix} \frac{1}{2} & 1 \end{pmatrix} \begin{pmatrix} s_{d,d} & s_{d,s} \\ s_{s,d} & s_{s,s} \end{pmatrix} \begin{pmatrix} \frac{1}{2} \\ 1 \end{pmatrix} = \frac{1}{4}s_{d,d} + s_{d,s} + s_{s,s}$ which gives $(s_{ii} - \boldsymbol{t}_i'\boldsymbol{S}_{i-1}\boldsymbol{t}_i)^{-1} = \left(1 - \frac{1}{4}s_{d,d} - s_{s,s}\right)^{-1} = \left(1 - \frac{1}{4}(1 + F_d) - (1 + F_s)\right)^{-1}$ where inbreeding coefficients $F_d = S_{d,d} - 1$ and $F_s = S_{s,s} - 1$ are defined for X-chromosomes (not for autosomes). This $\left(1 - \frac{1}{4}(1 + F_d) - (1 + F_s)\right)^{-1}$ is the value of $1/d$ above, and this coefficient is summed to locations indicated by $\begin{pmatrix} \boldsymbol{t}_i\boldsymbol{t}_i' & -\boldsymbol{t}_i \\ -\boldsymbol{t}_i' & 1 \end{pmatrix}$ which corresponds to the rules above. Note that we have defined here males' inbreeding coefficient as $F_s = S_{s,s} - 1$ and therefore males have an inbreeding of -0.5. Using the same method in males gives gives $(s_{ii} - \boldsymbol{t}_i'\boldsymbol{S}_{i-1}\boldsymbol{t}_i)^{-1} = 0.5 - \frac{1}{4}(1 + F_d)$ because the term in t_i corresponding to the sire of a male is 0.

The method applies equally well to relationships computed with metafounders, provided that the initial matrix is $\mathbf{\Gamma}^{-1}$ is invertible. Inbreeding of metafounders corresponds to the diagonal of $\mathbf{\Gamma}$ minus 1. A code is provided in Supplementary material.

Additional reference:

Derivation of rules for the inverse of the numerator relationship matrix: partitioned matrix approach. J Solkner and B Tier. Workshop on advanced biometrical methods in animal breeding, 9-15 October 1992, Flawil, Switzerland.
