# Supplementary material for: Theoretical and empirical comparisons of expected and realized relationships for the X-chromosome
Source: Genet Sel Evol. 2020 Aug 20;52:50. doi: 10.1186/s12711-020-00570-6 (PMC7441635; doi:10.1186/s12711-020-00570-6)
Supplement: Supplementary file 2 — Additional file 2: Table S1. Comparison of realized (marker-based) and expected (pedigree-based) additive genetic relationships on the X-chromosome (specific part) for different categories of animals. Table S2. Comparison of realized (marker-based) and expected (pedigree-based) additive genetic relationships on the autosomes (all together) for different categories of animals. Table S3. Comparison of realized (marker-based) and expected (pedigree-based) additive genetic relationships on the BTA2 for different categories of animals. [file 12711_2020_570_MOESM2_ESM.pdf]

Supp. Table 1. Comparison of realized (marker-based) and expected (pedigree-based) additive genetic relationships on the X-chromosome (specific part) for different categories of animals.

| Relationship class | Expected | Realized | SD | Median | Min | Max |
|---|---|---|---|---|---|---|
| Sire / son | 0.031 | -0.027 | 0.136 | -0.061 | -0.205 | 0.970 |
| Sire / daughter | 0.721 | 0.697 | 0.054 | 0.695 | 0.539 | 0.935 |
| Dam / son | 0.721 | 0.700 | 0.053 | 0.692 | 0.597 | 0.864 |
| Dam / daughter | 0.539 | 0.479 | 0.088 | 0.472 | 0.250 | 0.881 |
| Paternal half-sibs (two males) | 0.066 | 0.038 | 0.191 | -0.013 | -0.267 | 1.001 |
| Paternal half-sibs (male/female) | 0.064 | -0.021 | 0.146 | -0.053 | -0.321 | 0.799 |
| Paternal half-sibs (two females) | 0.546 | 0.479 | 0.102 | 0.468 | 0.181 | 1.003 |
| Maternal half-sibs (two males) | 0.522 | 0.523 | 0.256 | 0.520 | 0.041 | 1.000 |
| Maternal half-sibs (male/female) | 0.387 | 0.339 | 0.223 | 0.359 | -0.197 | 0.799 |
| Maternal half-sibs (two females) | 0.309 | 0.228 | 0.177 | 0.235 | -0.282 | 0.946 |
| Full-sibs (two males) | 0.510 | 0.416 | 0.277 | 0.410 | -0.021 | 0.972 |
| Full-sibs (male / female) | 0.383 | 0.315 | 0.216 | 0.289 | -0.223 | 0.787 |
| Full-sibs (two females) | 0.768 | 0.734 | 0.148 | 0.745 | 0.308 | 1.002 |
| Diagonal elements (males) | 1.000 | 1.000 | 0.000 | 1.000 | 1.000 | 1.000 |
| Diagonal elements (females) | 1.000 | 1.000 | 0.000 | 1.000 | 1.000 | 1.000 |

Supp. Table 2. Comparison of realized (marker-based) and expected (pedigree-based) additive genetic relationships on the autosomes (all together) for different categories of animals.

| Relationship class | Expected | Realized | SD | Median | Min | Max |
|---|---|---|---|---|---|---|
| Sire / son | 0.541 | 0.482 | 0.026 | 0.479 | 0.423 | 0.560 |
| Sire / daughter | 0.543 | 0.482 | 0.024 | 0.481 | 0.403 | 0.596 |
| Dam / son | 0.545 | 0.481 | 0.019 | 0.480 | 0.447 | 0.520 |
| Dam / daughter | 0.545 | 0.484 | 0.021 | 0.484 | 0.420 | 0.554 |
| Paternal half-sibs (two males) | 0.324 | 0.230 | 0.050 | 0.227 | 0.061 | 0.482 |
| Paternal half-sibs (male/female) | 0.324 | 0.225 | 0.045 | 0.223 | 0.065 | 0.453 |
| Paternal half-sibs (two females) | 0.322 | 0.226 | 0.043 | 0.225 | 0.044 | 0.475 |
| Maternal half-sibs (two males) | 0.318 | 0.229 | 0.054 | 0.229 | 0.124 | 0.353 |
| Maternal half-sibs (male/female) | 0.321 | 0.229 | 0.044 | 0.228 | 0.118 | 0.375 |
| Maternal half-sibs (two females) | 0.321 | 0.232 | 0.043 | 0.231 | -0.035 | 0.388 |
| Full-sibs (two males) | 0.544 | 0.476 | 0.056 | 0.480 | 0.328 | 0.548 |
| Full-sibs (male / female) | 0.546 | 0.477 | 0.047 | 0.479 | 0.327 | 0.587 |
| Full-sibs (two females) | 0.542 | 0.482 | 0.052 | 0.482 | 0.241 | 1.000 |
| Diagonal elements (males) | 1.000 | 1.000 | 0.000 | 1.000 | 1.000 | 1.000 |
| Diagonal elements (females) | 1.000 | 1.000 | 0.000 | 1.000 | 1.000 | 1.000 |

Supp .Table 3. Comparison of realized (marker-based) and expected (pedigree-based) additive genetic relationships on the BTA2 for different categories of animals.

| Relationship class | Expected | Realized | SD | Median | Min | Max |
|---|---|---|---|---|---|---|
| Sire / son | 0.541 | 0.470 | 0.074 | 0.463 | 0.299 | 0.758 |
| Sire / daughter | 0.543 | 0.474 | 0.080 | 0.470 | 0.237 | 0.906 |
| Dam / son | 0.545 | 0.462 | 0.079 | 0.460 | 0.300 | 0.700 |
| Dam / daughter | 0.545 | 0.476 | 0.079 | 0.473 | 0.225 | 0.804 |
| Paternal half-sibs (two males) | 0.324 | 0.218 | 0.175 | 0.218 | -0.342 | 0.829 |
| Paternal half-sibs (male/female) | 0.324 | 0.207 | 0.173 | 0.209 | -0.381 | 0.923 |
| Paternal half-sibs (two females) | 0.322 | 0.208 | 0.173 | 0.212 | -0.379 | 0.934 |
| Maternal half-sibs (two males) | 0.318 | 0.175 | 0.172 | 0.156 | -0.224 | 0.560 |
| Maternal half-sibs (male/female) | 0.321 | 0.210 | 0.174 | 0.200 | -0.285 | 0.959 |
| Maternal half-sibs (two females) | 0.321 | 0.222 | 0.172 | 0.217 | -0.340 | 0.716 |
| Full-sibs (two males) | 0.544 | 0.510 | 0.184 | 0.480 | 0.133 | 0.855 |
| Full-sibs (male / female) | 0.546 | 0.446 | 0.203 | 0.442 | -0.098 | 0.882 |
| Full-sibs (two females) | 0.542 | 0.473 | 0.212 | 0.481 | -0.279 | 1.000 |
| Diagonal elements (males) | 1.000 | 1.000 | 0.000 | 1.000 | 1.000 | 1.000 |
| Diagonal elements (females) | 1.000 | 1.000 | 0.000 | 1.000 | 1.000 | 1.000 |
