# Supplementary figures and images for: Theoretical and empirical comparisons of expected and realized relationships for the X-chromosome
Source: Genet Sel Evol. 2020 Aug 20;52:50. doi: 10.1186/s12711-020-00570-6 (PMC7441635; doi:10.1186/s12711-020-00570-6)

**All genotyped individuals**

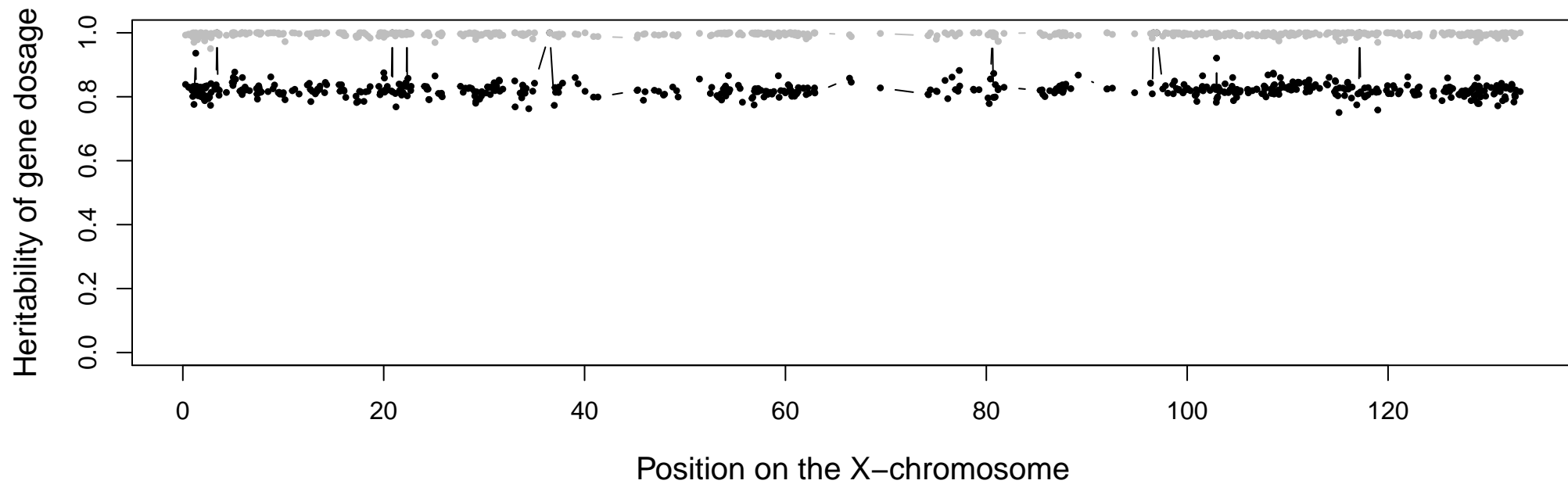

**Genotyped males**

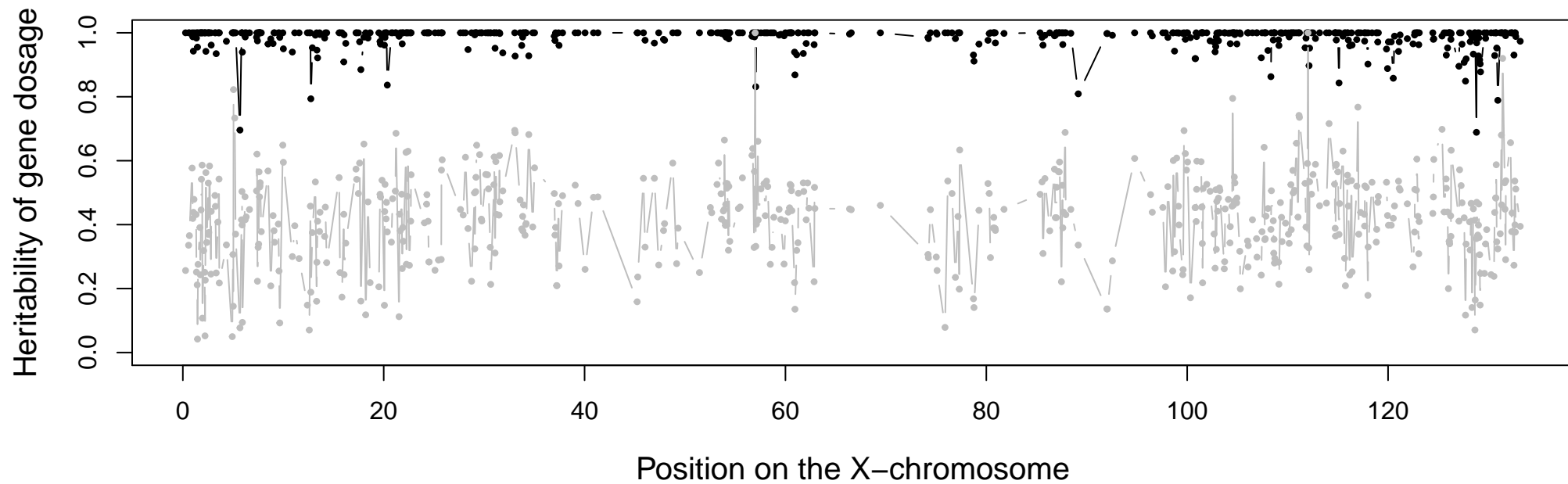

Supplement: Supplementary file 3 — Additional file 3: Figure S1. Heritability of the gene content along the X-chromosome (X-specific part), when males considered homozygous are coded as {0,2}. A. For all genotyped individuals, B. For genotyped males only. Black and gray dots indicate heritabilities estimated with the pedigree-based relationships using rules specific to the X-chromosome (S) and general rules for the autosomes (A), respectively. [file 12711_2020_570_MOESM3_ESM.pdf]
